# Real-Time Measurement of Refractive Index Using 3D-Printed Optofluidic Fiber Sensor

**DOI:** 10.3390/s22239377

**Published:** 2022-12-01

**Authors:** João M. Leça, Yannis Magalhães, Paulo Antunes, Vanda Pereira, Marta S. Ferreira

**Affiliations:** 1 i3N & Physics Department, University of Aveiro, Campus Universitario de Santiago, 3810-193 Aveiro, Portugal; 2 ISOPlexis—Sustainable Agriculture and Food Technology Center, University of Madeira, Campus da Penteada, 9020-105 Funchal, Portugal

**Keywords:** optofluidics, Fabry–Perot, refractive index, real-time measurement

## Abstract

This work describes a 3D-printed optofluidic fiber sensor to measure refractive index in real time, combining a microfluidic system with an optical fiber extrinsic Fabry–Perot interferometer. The microfluidic chip platform was developed for this purpose through 3D printing. The Fabry–Perot cavity was incorporated in the microfluidic chip perpendicularly to the sample flow, which was of approximately 3.7 µL/s. The optofluidic fiber sensor platform coupled with a low-cost optical power meter detector was characterized using different concentrations of glucose solutions. In the linear regression analysis, the optical power shift was correlated with the refractive index and a sensitivity of −86.6 dB/RIU (r^2^ = 0.996) was obtained. Good results were obtained in terms of stability with a maximum standard deviation of 0.03 dB and a sensor resolution of 5.2 × 10^−4^ RIU. The feasibility of the optofluidic fiber sensor for dynamic analyses of refractive index with low sample usage was confirmed through real-time measurements.

## 1. Introduction

Optofluidics is a research and technology area that combines elements of optics and photonics into microfluidic devices. This sensing technology is based upon detecting changes in the optical properties to characterize the flowing media [1,2]. Optofluidic sensing platforms are typically composed of microfluidic on-chip waveguides and off-chip optical components such as light sources and detectors [2]. Incorporating optical fiber sensors in optofluidics platforms can lead to a powerful tool that simplifies analyses when compared to traditional analytical methods, such as wet chemistry techniques, spectroscopy, spectrometry, and chromatography. Optical fiber sensors continue to attract the attention of researchers because of their compactness, lightweight, fast-response, eco-friendliness, immunity to electromagnetic interference, and remote sensing capability with the possibility of the sensor being distant from the acquisition and processing systems [3,4,5,6].

The accurate measurement of liquid refractive Index is important in various scientific and industrial fields, including food and beverages (e.g., wine fermentation) and pharmaceutical industries (e.g., chemical reactions), process control, quality, and safety. Typically, samples are collected for refractive index measurements in refractometric devices, but distinct efforts have been made to evaluate this parameter in situ and remotely. A wide variety of configurations based on optical fiber sensors for the measurement of refractive index have been reported, such as Fabry–Perot interferometers [7], multimode interferometers [8], long period gratings [9], fiber Bragg gratings [10], fiber tapers [11], and microstructured fibers [12].

Most of the optical fiber sensors for refractive index measurement are based on the creation of specific optical fiber tips that can be dipped or held in liquid solutions. The fiber tip-interaction concept has been explored in different ways such as a multimode fiber section spliced at the end of a single-mode fiber [13], a suspended core between two single-mode fibers [14], a sensing head with a fiber Bragg grating and a Fabry–Perot cavity obtained with a thin film coating [15], an open micro-hole with an excimer laser [16], and a fiber tip structure formed with two-photon polymerization lithography [7], among other approaches [17].

In terms of optofluidic platforms for refractive index analysis, different microfluidic systems, geometries, materials, and approaches have been used to incorporate the optical fiber sensors. St-Gelais et al. [18] used vertically etched silicon Bragg reflectors to fabricate an integrated Fabry–Perot cavity simultaneously with the microfluidic system. Later, a microfluidic sensor based on an all-silica in-line interferometer was fabricated with a silica tube sandwiched between two microstructured optical fibers [19]. Duduś et al. [20] developed a microfluidic chip with a curable photopolymer with two attachable sections, allowing liquids to be pumped through the channels into the Mach–Zehnder interferometer setup. An in-line optofluidic sensor in a side-channel photonic crystal fiber spliced with side-polished single-mode fibers was also developed, combining a long-period grating with an intermodal interference [21]. None of these works explored the 3D printing technique to develop the optofluidic sensing platform.

Usually, optofluidic sensors are employed in biological or chemical applications, through the measurement of different parameters such as absorption, scattering, reflection, refraction, and plasmonics [22]. As for the chip manufacturing, different techniques have been explored, from UV lithography, stereolithography, multi jet modeling, 3D printing, among others. A compromise is usually required, between cost, resolution, and degrees of freedom in the produced structures. Different reviews have been reported regarding this type of sensors, as it is a very active field of research [22,23,24].

In this work, a new optofluidic fiber sensor is proposed. To the best of our knowledge, 3D printing technology was used for the first time to design and create a microfluidic platform for the real-time measurement of refractive index, remotely and without sample collection using a low-cost interrogation system. This was achieved using a fiber-optic extrinsic Fabry–Perot interferometer connected to a microfluidic system, combining the intrinsic advantages of microfluidic systems and optical fiber sensors.

## 2. Materials and Methods

### 2.1. Optofluidic Platform Fabrication and Configuration

**Figure 1:**
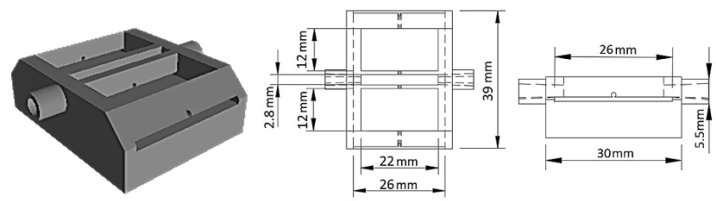
Microfluidic chip design and dimensions.

The fabrication of the optofluidic platform started with the design of the microfluidic chip using the Autodesk Fusion 360^®^ 2.0.14106 software (San Francisco, CA, USA), as shown in Figure 1. The chip was 3D printed in a BCN3D Sigma R19 3D printer (Barcelona, Spain) using an Acrylonitrile Butadiene Styrene (ABS) thermoplastic and amorphous polymer. The printing density was of 20% and the layer height was of 0.20 mm. The cylindrical protuberances on the side surfaces were designed to provide support to the microfluidic tubes. These have an external diameter of 5.5 mm that funnels to 2.8 mm, allowing the fitting of the Tygon^®^ tubes (1 mm inner diameter). The side tube fittings are directly connected to the measurement cavity that has a geometric shape of a rectangular prism, ABS side barriers, and is open on the top. As can be seen in the 3D schematic design and front view of the technical design in Figure 1, a gap was left to place a laboratory glass slide and a hole to pass the optical fiber. The incorporated glass microscope slide was used as a support for the optical fiber, not only to facilitate the fiber alignment and fixation, but also for minimizing relaxation and thermal expansion effects of the polymeric material, which could compromise the sensor response. During the experiments, it was verified that directly attaching the sensor to the polymer did not provide the stability necessary to the interferometric sensor signal.

**Figure 2:**
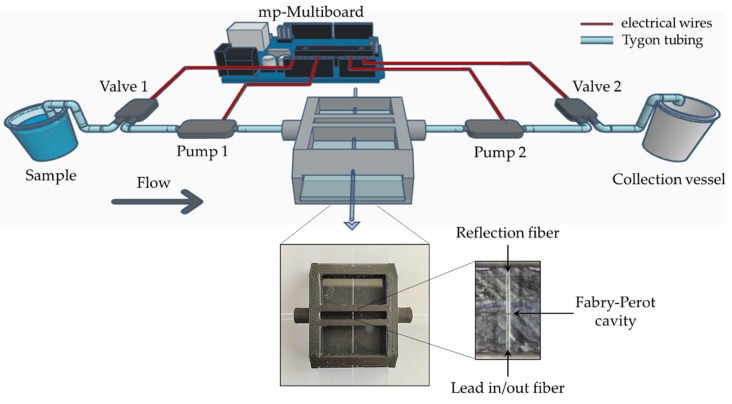
Experimental scheme of the optofluidic platform. The inset shows a photograph of the 3D-printed optofluidic fiber sensor.

Figure 2 presents the schematic diagram of the optofluidic setup. The 3D printed microfluidic chip was connected to a microfluidic kit (Darwin Microfluidics, Paris, France), composed by 2 pumps and 2 valves controlled by a Bartels mp-Multiboard micropump setup incorporated with an Arduino Pro Micro microcontroller. Pump 1 conducts the sample to the microfluidic chip cavity with the optical fiber sensor, where the light interacts with the flowing sample, whereas pump 2 leads the sample to a collection vessel. Both pumps were adjusted so that the inlet and the outlet liquid flow rates were equaled, and the sample volume was maintained in the measurement cavity. The flow rate was approximately 3.7 µL/s and could be adjusted as long as it was ensured that the liquid flow was laminar and constant in the Fabry–Perot cavity to perform the dynamic measurements.

To fabricate the sensing Fabry–Perot cavity, two commercial single-mode fibers (SMF28, from Corning, NY, USA) were stripped, cleaved with an optical fiber high-precision cutting machine, inserted one on each side of the microfluidic chip, and aligned inside of the chip cavity using a digital camera with optical zoom. After the alignment, both fibers were fixed to the glass slide incorporated in the microfluidic chip with UV curing resin, ensuring an immediate adhesion and preventing resin from mis-aligning the fibers or even moving to critical sensor locations, such as the fiber tip. One of the fibers was connected to the interrogation setup, and the other was only used as reflective material.

### 2.2. Experimental Setup

The broadband optical source (Amonics LS—CL-17-B-FA, Hong Kong) used in the experiments has a center wavelength of 1570 nm and a bandwidth of 80 nm. The sensor was firstly interrogated with an optical spectrum analyzer (OSA, Anritsu MS9740A, Kanagawa, Japan), where spectra were recorded from 1530 to 1610 nm. The OSA, together with the digital camera with optical zoom, were combined to align the cavity and optimize the characteristics of the reflected signal. The analysis could have continued with the OSA, exploring its greater sensitivity and the possibility of spectral analysis, but our aim was to develop a low-cost approach. Thus, the detector was replaced by a FOPM-203 handheld optical power meter (FS, Neufahrn, Germany) set at 1550 nm, with a resolution of 0.01 dB, to validate the optofluidic fiber sensor for the real-time measurement of refractive index. Notice that the cost may be even further decreased if an LED replaces the optical source and a photodetector connected with a data acquisition system (DAQ) replaces the optical power meter. This can also enhance the portability of the sensing structure and may increase the acquisition rate and the application in different environments.

**Figure 3:**
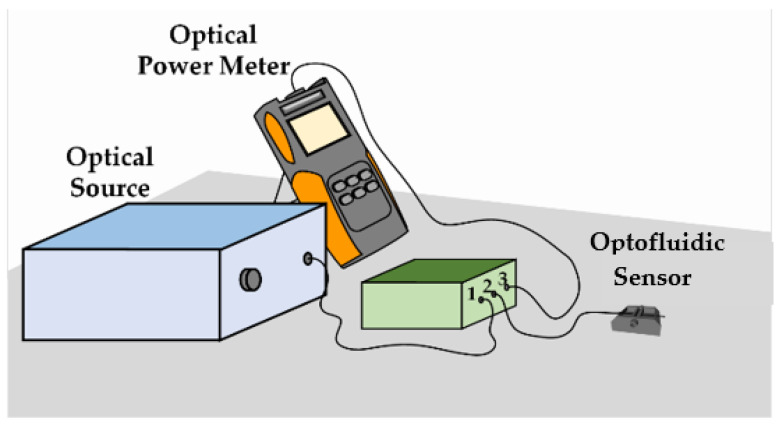
Schematic diagram of experimental setup.

The optofluidic sensor was connected to the optical circulator in a typical reflection scheme (Figure 3), with the optical source in port 1, the Fabry–Perot interferometric sensor in port 2, and the optical power meter in port 3. In the dynamic real-time measurements, the optical power was recorded every second. All analyses were performed at room temperature, at about 24 °C.

### 2.3. Glucose Solutions

To perform this study, different D-(*+*)-glucose (José Manuel Gomes dos Santos, Odivelas, Portugal) solutions were prepared in deionized water. With an analytical scale, a solution of 40 wt.% was carefully prepared. From this more concentrated solution, working solutions were prepared with concentrations ranging from 0 to 40 wt.%, with intervals of 5 wt.%, by successive dilutions with deionized water. The refractive indices of these samples were extrapolated according to Pereira et al. [25] because of the fact that the interrogation setup was set at 1550 nm. To confirm that the solutions were correctly prepared, the refractive indices were also measured in a refractometer Easy R40 (Mettler Toledo, Columbus, OH, USA).

## 3. Experiment Results and Discussion

**Figure 4:**
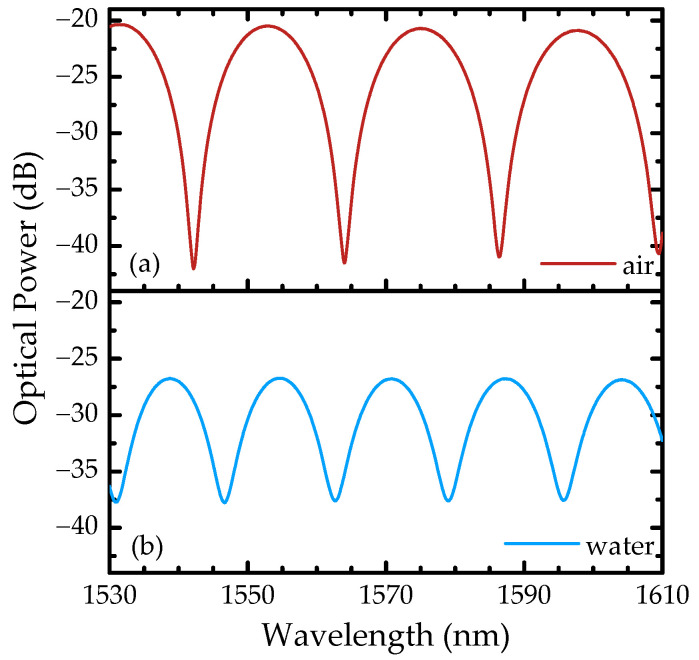
Sensor spectra in (**a**) air and (**b**) water of the fiber-optic extrinsic Fabry–Perot interferometer recorded from 1530 to 1610 nm in the optical spectrum analyzer.

Figure 4 presents the reflection spectra in air and water, recorded from 1530 to 1610 nm, after the alignment of the optical fibers and before changing to the optical power meter interrogation system, where it is possible to observe the characteristic interferometric pattern of a Fabry–Perot two-wave interferometer. Considering the wavelengths of two adjacent peaks in air, *λ*_1_ and *λ*_2_, a cavity length of *L*_FP_ = 56 μm was estimated using the following equation:

(1) $$\Delta \lambda =\frac{\lambda_{1}\lambda_{2}}{2n_{eff}L_{FP}}$$

where ∆*λ* = *λ*_2_ − *λ*_1_. It was also possible to determine that the air and water spectra have a visibility of 98% and 85%, which is related to the decrease on the reflectivity in each interface as the cavity refractive index increases. Furthermore, as the refractive index increases, so do the optical losses, as shown in Figure 4.

**Figure 5:**
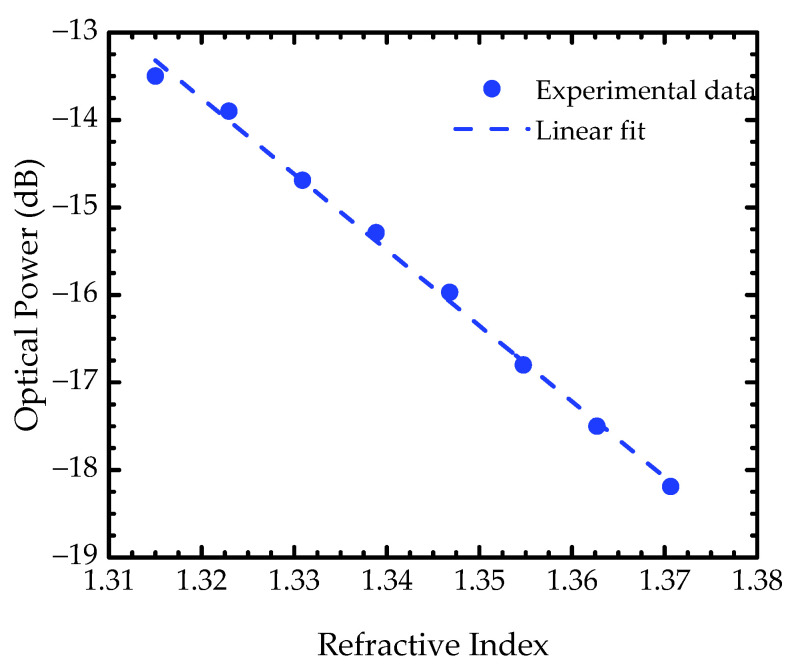
Experimental results of the optical power response to the correspondent refractive indices. The blue circles are the obtained data and the dashed blue line the applied linear fit.

To evaluate the analytical behavior against refractive index, the sensor was exposed to different concentrations of glucose, and the optical power was recorded at every concentration, from 0 to 40 wt.% of glucose. As previously observed, as the solution refractive index increases, the optical power decreases. This is mainly related to a decrease in the interface’s reflectivity. A linear fitting was applied to the entire dynamic range to correlate the optical power response with the corresponding refractive indices of the glucose samples (Figure 5). A sensitivity of −86.6 dB/RIU (r^2^ = 0.996) was obtained, which corresponds to the slope of the linear fit, being in the same order of magnitude of the values found in literature (from −62 to −205 dB/RIU) [13,15,26], with the advantage of using a low-cost detector as an alternative to the optical spectrum analyzer typically used.

**Figure 6:**
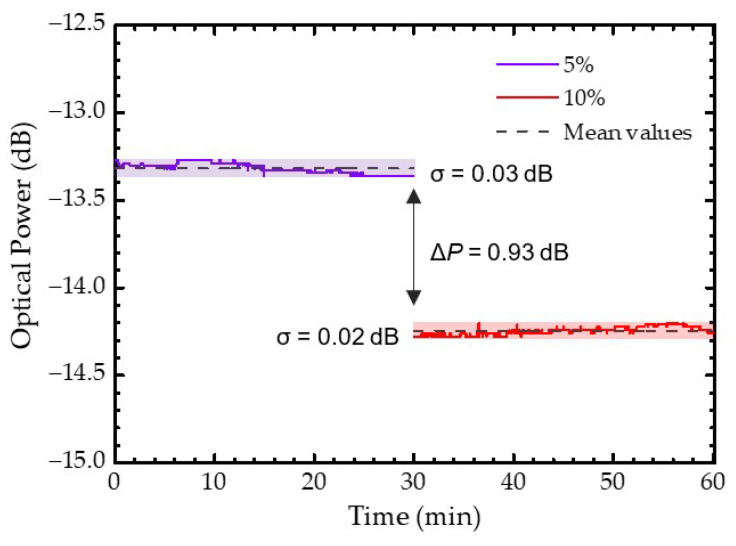
Optical power stability over a period of 60 min for 5 wt.% and 10 wt.% of glucose solutions. The shadowed zones represent the standard deviation (*σ*) of each concentration.

To determine the optofluidic fiber sensor stability and resolution, the optical power response was analyzed over 60 min (Figure 6), for the concentration steps of 5 wt.% and 10 wt.% of glucose, individually. These measurements were made with an approximated flow rate of 3.7 µL/s and the optical power was recorded every second. One could observe a good stability, with a maximum standard deviation of 0.03 dB, which is close to the equipment resolution. Optical power mean values of −13.32 and −14.25 dB were obtained for the 5 wt.% and 10 wt.% steps, respectively, and a resolution value (*δn*) of 5.2 × 10^−4^ RIU was calculated through the following equation [27]:

(2) $$\delta n=\frac{2\sigma_{p}\Delta n}{\Delta P}.$$

The *σ_P_* corresponds to the maximum optical power standard deviation, the Δ*n* is the variation in refractive index between each step and Δ*P* is the variation in optical power. The resolution obtained is comparable to the most recent refractometric devices and sometimes comparable to the Fabry–Perot sensors that used an OSA as interrogation system, which is a more expensive equipment [13,15,26].

**Figure 7:**
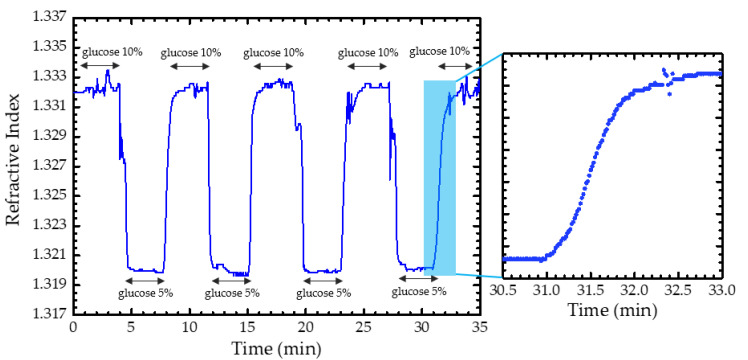
Optofluidic fiber sensor real-time measurement of refractive index in transitions between samples of 5 wt.% and 10 wt.% of glucose.

In the last phase of this study, we verified the feasibility of the optofluidic fiber sensor for dynamic analyses in real time with its characteristic low sample volume usage. These analyzes were also made in an air-conditioned room at about 24 °C with an approximated flow rate of 3.7 µL/s and the optical power recorded every second. The solutions chosen for this experimental part of the study were also the 5 wt.% and 10 wt.% of glucose, since these are the less concentrated glucose solutions of the calibration range. Four transition cycles between the chosen solutions (Figure 7) were performed, and it was possible to verify that the sensor was robust for real-time analyses. The dynamic transition between samples can be monitored with this optofluidic sensor, and it requires an average of approximately 50 s to stabilize again. This is mainly related to the time required for the solution to be exchanged effectively in the optofluidic chip. Furthermore, the spikes observed in the transition between the higher and lower concentrations can be related to the non-uniformity of the solution’s refractive index during this step. This result allows us to verify that this sensor can be applied in studies of molecular diffusions and solutions or reactions microenvironments.

## 4. Conclusions

In conclusion, a 3D-printed optofluidic fiber sensor was successfully developed for refractive index measurements in real time, combining a microfluidic system with a fiber-optic extrinsic Fabry–Perot interferometer. This sensor does not require sample pre-treatment and uses small volumes, minimizing sample manipulation and increasing its control during measurements. The optofluidic fiber sensor platform was coupled to a low-cost power meter detector, and good results were obtained with a sensitivity of −86.6 dB/RIU, a maximum standard deviation of 0.03 dB (over 60 min), and a sensor resolution value of 5.2 × 10^−4^ RIU. The easy production, reduced footprint, electric passivity, and linear, stable, and sensitive instantaneous response make this sensor a strong candidate for dynamic and accurate real-time measurement of the refractive index in liquids. Besides its potential for general applications in food and beverages and pharmaceutical industries, it can be explored to study molecular diffusions and reactions or microenvironments.

## References

[1] Blue R., Duduś A., Uttamchandani D. (2016). A review of single-mode fiber optofluidics. IEEE J. Sel. Top. Quantum Electron..

[2] Psaltis D., Quake S.R., Yang C. (2006). Developing optofluidic technology through the fusion of microfluidics and optics. Nature.

[3] Danchuk A.I., Komova N.S., Mobarez S.N., Doronin S.Y., Burmistrova N.A., Markin A.V., Duerkop A. (2020). Optical sensors for determination of biogenic amines in food. Anal. Bioanal. Chem..

[4] Umapathi R., Park B., Sonwal S., Rani G.M., Cho Y., Huh Y.S. (2022). Advances in optical-sensing strategies for the on-site detection of pesticides in agricultural foods. Trends Food Sci. Technol..

[5] Seki A., Narita K., Watanabe K. (2016). Refractive index measurement in sucrose solution and beverage using surface plasmon resonance sensor based on hetero-core structured fiber optic. Procedia Chem..

[6] Paixão T., Nunes A.S., Bierlich J., Kobelke J., Ferreira M.S. (2022). Fabry-Perot interferometer based on suspended core fiber for detection of gaseous ethanol. Appl. Sci..

[7] Cao S., Shang X., Yu H., Shi L., Zhang L., Wang N., Qiu M. (2022). Two-photon direct laser writing of micro Fabry-Perot cavity on single-mode fiber for refractive index sensing. Opt. Express.

[8] Novais S., Ferreira C.I.A., Ferreira M.S., Pinto J.L. (2018). Optical fiber tip sensor for the measurement of glucose aqueous solutions. IEEE Photon. J..

[9] Esposito F., Ranjan R., Campopiano S., Iadicicco A. (2017). Experimental study of the refractive index sensitivity in arc-induced long period gratings. IEEE Photon. J..

[10] Yang M., Dai J., Li X., Wang J. (2010). Side-polished fiber Bragg grating refractive index sensor with TbFeCo magnetoptic thin film. J. Appl. Phys..

[11] Yadav T.K., Narayanaswamy R., Abu Bakar M.H., Kamil Y.M., Mahdi M.A. (2014). Single mode tapered fiber-optic interferometer based refractive index sensor and its application to protein sensing. Opt. Express.

[12] Jha R., Villatoro J., Badenes G. (2008). Ultrastable in reflection photonic crystal fiber modal interferometer for accurate refractive index sensing. Appl. Phys. Lett..

[13] Silva S., Frazão O., Santos J.L., Malcata F.X. (2012). A reflective optical fiber refractometer based on multimode interference. Sens. Actuators B Chem..

[14] Frazão O., Baptista J.M., Santos J.L., Kobelke J., Schuster K. (2009). Refractive index tip sensor based on Fabry-Perot cavities formed by a suspended core fibre. J. Eur. Opt. Soc. Rapid Publ..

[15] Chen L.X., Huang X.G., Li J.Y., Zhong Z.B. (2012). Simultaneous measurement of refractive index and temperature by integrating an external Fabry-Perot cavity with a fiber Bragg grating. Rev. Sci. Instrum..

[16] Zhang W., Liu Y., Zhang T., Yang D., Wang Y., Yu D. (2019). Integrated fiber-optic Fabry-Pérot interferometer sensor for simultaneous measurement of liquid refractive index and temperature. IEEE Sens. J..

[17] Zheng Y., Chen L.H., Yang J., Raghunandhan R., Dong X., So P.L., Chan C.C. (2017). Fiber optic Fabry–Perot optofluidic sensor with a focused ion beam ablated microslot for fast refractive index and magnetic field measurement. IEEE J. Sel. Top. Quantum Electron..

[18] St-Gelais R., Masson J., Peter Y.-A. (2009). All-silicon integrated Fabry–Pérot cavity for volume refractive index measurement in microfluidic systems. Appl. Phys. Lett..

[19] Tian J., Lu Y., Zhang Q., Han M. (2013). Microfluidic refractive index sensor based on an all-silica in-line Fabry–Perot interferometer fabricated with microstructured fibers. Opt. Express.

[20] Duduś A., Blue R., Uttamchandani D. (2013). Comparative study of microfiber and side-polished optical fiber sensors for refractometry in microfluidics. IEEE Sens. J..

[21] Zhang N., Humbert G., Wu Z., Li K., Shum P.P., Zhang N.M.Y., Cui Y., Auguste J.-L., Dinh X.Q., Wei L. (2016). In-line optofluidic refractive index sensing in a side-channel photonic crystal fiber. Opt. Express.

[22] Tang J., Qiu G., Wang J. (2022). Recent development of optofluidics for imaging and sensing applications. Chemosensors.

[23] Bhattacharjee N., Urrios A., Kang S., Folch A. (2016). The upcoming 3D-printing revolution in microfluidics. Lab Chip.

[24] Santos D.M., Cardoso R.M., Migliorini F.L., Facure M.H.M., Mercante L.A., Mattoso L.H.C., Correa D.S. (2022). Advances in 3D printed sensors for food analysis. TrAC Trends Anal. Chem..

[25] Pereira D., Bierlich J., Kobelke J., Ferreira M.S. (2022). Hybrid sensor based on a hollow square core fiber for temperature independent refractive index detection. Opt. Express.

[26] Zhao J.R., Huang X.G., He W.X., Chen J.H. (2010). High-resolution and temperature-insensitive fiber optic refractive index sensor based on fresnel reflection modulated by Fabry–Perot interference. J. Light. Technol..

[27] Romero R., Frazão O., Pereira D.A., Salgado H.M., Araújo F.M., Ferreira L.A. (2005). Intensity-referenced and temperature-independent curvature-sensing concept based on chirped fiber Bragg gratings. Appl. Opt..

